# Artificial Intelligence in Foodborne Pathogen Detection from Sensing to Food Safety Systems: A Systematic Review

**DOI:** 10.3390/foods15142562

**Published:** 2026-07-21

**Authors:** Maria Schirone, Giovanni D’Ambrosio, Antonello Paparella

**Affiliations:** Department of Bioscience and Technology for Food, Agriculture and Environment, University of Teramo, Via Balzarini 1, 64100 Teramo, Italy; mschirone@unite.it (M.S.); gdambrosio@unite.it (G.D.)

**Keywords:** machine learning, predictive analytics, validation, food safety governance, regulatory framework, artificial intelligence

## Abstract

This systematic review summarises advances in artificial intelligence (AI) and machine learning (ML) for foodborne pathogen detection, covering applications in various technologies (AI-assisted microscopy, spectroscopy, biosensors and sensor-based systems), food supply chains, analytical performance, operational metrics and regulatory developments, addressing gaps in previous reviews limited to individual technologies or lacking regulatory analysis. Following PRISMA 2020 guidelines, Scopus, PubMed, and Web of Science were searched from 1 January 2010 to 25 June 2026 using a validated string. Inclusion criteria were explicit detection of a pathogen, clearly described AI/ML algorithm, study evaluation on food or supply chains, and quantitative validation metrics. Exclusion criteria were chemical-only studies, human-diagnostic studies, or purely theoretical studies. Given heterogeneity in the evidence, qualitative quality indicators were favoured over formal quantitative risk-of-bias tools, in distinction to internal cross-validation versus independent external validation. Key data were extracted using a standardised matrix, and after screening and snowballing, the final corpus consisted of 152 studies. CNN (Convolutional Neural Network)-based microscopy provides >99% accuracy in bacterial identification, SERS (Surface-Enhanced Raman Spectroscopy) and CNN 98.68% for pathogens and 99.85% for resistant strains. ML-driven biosensors show 80–100% prediction accuracy in the presence of environmental noise. Yet, performance drops dramatically on external validation, with models falling from 95% internal to 78–82% on independent test sets. Supply chain applications cover meat, dairy, seafood and produce, but most are still at pilot scale. The main constraints are data heterogeneity, lack of public benchmarks, matrix interference, non-standard validation protocols, and regulatory dissonance. However, the integration of AI with Internet of Things (IoT), blockchain and edge computing improves sensitivity, reduces false results and enables real-time monitoring despite the challenges. AI is a powerful decision-support tool that complements existing food safety controls rather than replacing them. To translate these technologies reliably into routine practice, effective implementation requires rigorous external validation and regulatory harmonisation.

## 1. Introduction

Foodborne diseases represent a major global public health challenge, which needs effective monitoring and prevention strategies [1]. According to the latest estimates released by the World Health Organisation (WHO) on the World Food Safety Day, foodborne diseases account for approximately 866 million cases and 1.5 million deaths annually [2]. Although the overall burden of foodborne diseases has decreased since 2000, severe regional inequalities persist, with the highest number of cases occurring in Africa and South-East Asia.

Traditional microbiological detection methods, while reliable, are time-consuming, labour-intensive, and often require several days to provide actionable results [3]. This creates significant challenges for real-time food safety monitoring and outbreak prevention. Artificial intelligence (AI) and machine learning (ML) technologies have emerged as valuable tools to address these limitations by enabling rapid and accurate pathogen detection while facilitating the analysis of complex microbiological datasets [4].

AI lacks a universally accepted definition. Collins and colleagues [5] identified 28 distinct definitions in a systematic review of the scientific literature. However, regarding food safety, AI can be defined as “a field of research in computer science that focuses on developing and studying methods and software that enable machines to perceive their environment, learn from it, and take intelligent actions to maximise the chances of achieving defined goals” [6,7].

ML, a major subfield of AI, refers to a broad range of algorithms that perform predictions based on data [8] and typically identify patterns between variables in large datasets [9]. ML algorithms improve predictive performance through iterative optimisation of model parameters using training data [10]. The integration of AI and ML software with conventional analytical methods has substantially improved the speed and cost-effectiveness of detecting toxic compounds of both chemical and biological origins [11]. AI and ML technologies address these limitations by enabling automated, objective analysis of morphological features thereby reducing subjectivity and improving analytical consistency [12].

Deep learning (DL) is a more advanced form of ML, with layers of algorithms and decision networks that progressively extract increasing levels of information [13]. Different definitions have been proposed for DL, among which the most comprehensive is the one given by Deng and Yu [14]: “a class of ML techniques that exploit many layers of non-linear information processing for supervised or unsupervised feature extraction and transformation, and for pattern analysis and classification”.

All these systems leverage advanced algorithms to extract meaningful patterns from complex, high-dimensional data generated by diverse sensing modalities. The convergence of AI-enabled signal processing with advanced biosensing technologies has created opportunities for real-time analytics, improved sensitivity, and multiplexed detection of foodborne pathogens [15].

Existing reviews have addressed some aspects of AI applications to food safety. One of the earliest reviews, published by Vilne et al. [16], focused on AI and ML use for epidemiological investigations of foodborne outbreaks. These authors analysed different technical issues regarding whole-genome sequencing analysis pipelines, such as genome assembly, comparative genomics, and phylogenomics, and reviewed the impact on pathogen detection and source tracing. Deng et al. [17] reviewed ML implementation using foodborne pathogen genomes for antimicrobial resistance prediction and source attribution, considering also the exploitation of novel data streams such as transactional and trade data analysis. Recently, Senmiao et al. [18] have reviewed AI applications to foodborne pathogen detection, considering also traceability and prevention and including a SWOT analysis for comparing different AI models. Onyeaka et al. [19] and Priyadharsshini and Adeyeye [20] examined the application of DL models and AI-enhanced biosensors, both possibly integrated with the Internet of Things (IoT) and blockchain, for real-time monitoring of foodborne pathogens. In the same field, Digital Twin (DT) technology has been recently reviewed as a method for integrating AI, IoT, and blockchain data for bacterial pathogen detection throughout the food supply chain [21]. Mohammadi et al. [22] reviewed the potential of AI-spectral technologies for food safety and focused their attention on the key limitations for technological transfer. Finally, Salaris et al. [23] published a systematic review on foodborne event prediction through social media mining, using Yelp and Twitter as main data sources combined with DL models for outbreak surveillance.

To the best of our knowledge, no review has covered at the same time the different technologies available for foodborne pathogen detection, e.g., AI-assisted microscopy techniques, spectroscopic methods, and sensor-based systems, considering the applications in different food production environments. Moreover, critical gaps in previous reviews refer to the regulatory framework, which has undergone significant changes in recent years. Therefore, this review aims to synthesise recent advances in AI and ML for foodborne pathogen detection, examining theoretical foundations, practical applications of different technologies in food supply chains, analytical performance, and regulatory developments. It also identifies current limitations and future research priorities for the implementation of AI-assisted food safety systems.

## 2. Method

### 2.1. Search Strategy and Screening

This review was not registered and did not receive any external funding. It was conducted according to the guidelines established by the Preferred Reporting Items for Systematic Reviews and Meta-analyses (PRISMA) to ensure the quality of the systematic review [24]. Firstly, the main issues of the topic were identified, including AI and ML applications regarding quality control, food microbiology applications and food chemistry, their applications in the food industry, food safety in the fifth industrial revolution, and the evolving regulatory framework on the matter. Secondly, relevant publications being published from 1 January 2010 to 25 June 2026 were retrieved from different search engines (Scopus, PubMed, and Web of Science), using the following validated search string:

TITLE-ABS-KEY ((“artificial intelligence” OR “machine learning” OR “deep learning” OR “neural network*” OR “CNN” OR “convolutional” OR “random forest” OR “support vector machine” OR “SVM” OR “ensemble” OR “transfer learning” OR “computer vision” OR “natural language processing”) AND (“foodborne pathogen*” OR “food-borne pathogen*” OR “bacterial detection” OR “microbial detection” OR “pathogen detection” OR “food contamination” OR “food safety” OR “Listeria” OR “Salmonella” OR “Escherichia coli” OR “Campylobacter”) AND (“spectroscopy” OR “biosensor*” OR “microscopy” OR “hyperspectral” OR “Raman” OR “imaging” OR “sensor” OR “PCR” OR “CRISPR” OR “HACCP”)) AND (LIMIT-TO (DOCTYPE, “ar”) OR LIMIT-TO (DOCTYPE, “re”)) AND (LIMIT-TO (LANGUAGE, “English”))

Boolean operators were applied to combine terms related to AI/ML (e.g., “artificial intelligence” and “machine learning”) with terms related to microbiological hazards (e.g., “food safety” and “foodborne pathogens”) and detection technologies (e.g., “spectroscopy” and “biosensors”). Two independent reviewers (A.P. and G.D.) conducted the title/abstract and full-text screening using the Rayyan QCRI web application to gain blinded and independent decision-making. Conflicts at either stage were resolved by consensus discussion. Where agreement could not be reached, a third author (M.S.) adjudicated the final decision.

### 2.2. Inclusion and Exclusion Criteria

Inclusion criteria required that the papers: 1. explicitly address the detection, quantification or identification of foodborne pathogens; 2. use a clearly described AI/ML algorithm as the primary data interpretation engine; 3. explicitly evaluate the technology on foods or beverages and/or in the supply chain; 4. report at least one quantitative metric useful for diagnostic validation. Exclusion criteria ruled out: 1. papers focused exclusively on chemical contaminants or quality parameters without connection with foodborne pathogens; 2. studies regarding exclusively human diagnostics; 3. publications using only basic descriptive statistics without a clear ML framework; 4. purely theoretical proposals; 5. conference abstracts, book chapters without original data, and editorials.

### 2.3. Data Extraction, Quality Appraisal, and Bias Considerations

A standardised extraction matrix was developed in Microsoft Excel to capture and organise key data from each eligible study, including AI method, detection mode, pathogen, food type, reported performance, and validation (e.g., cross-validation vs. external validation). Two independent authors (A.P. and G.D.) extracted data and compared them, with any discrepancies being resolved through discussion. Where agreement could not be reached, a third author (M.S.) adjudicated the final decision.

Eligible outcomes were broadly categorised as follows:

- Microscopy and image analysis;
- Spectroscopic detection;
- Biosensors;
- Food supply chains;
- Performance and metrics;
- Regulatory issues.

The following data were collected:

- The report: Author, year, and source of publication;
- The study: Sample size, matrix interference, and microorganisms;
- The research design and characteristics: Computational costs, type of validation, gaps and limits.

Given the heterogeneity of the evidence obtained, ranging from laboratory-scale studies to industrial pilot applications, a formal quantitative risk-of-bias assessment (e.g., QUADAS-2) was considered inappropriate and incompatible with the objective of mapping the emerging AI applications on foodborne pathogens. Instead, to ensure analytical rigour, qualitative quality indicators were incorporated into the synthesis, for instance by distinguishing studies that relied only on internal cross-validation and those in which independent external validation was applied.

### 2.4. Data Synthesis

**Figure 1:**
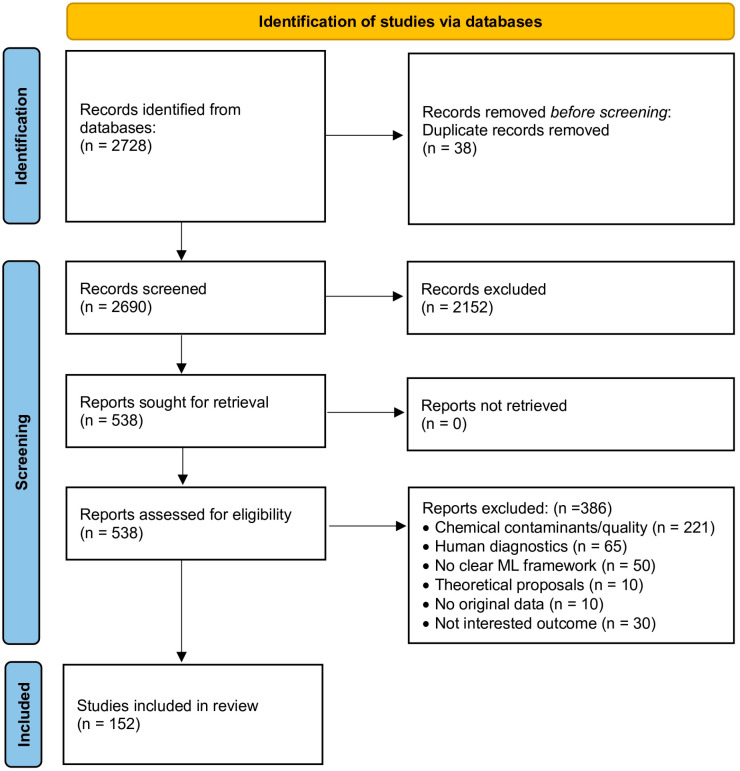
Flowchart of the literature search based on PRISMA.

The manuscripts were downloaded from different databases, including platforms such as Google Scholar and ResearchGate. All sources were last searched on 11 July 2026. Additional grey literature, including governmental (e.g., Centres for Disease Control and Prevention [CDC], WHO, and European Food Safety Authority [EFSA]) and industry reports, was also identified by targeted domain searches of regulatory archives. After duplicate removal, title/abstract screening, and full-text eligibility assessment, forward–backward snowballing was carried out to identify additional studies by searching the reference lists of publications eligible for full-text review and using Scopus to identify and screen studies citing them. Conference proceedings were consulted by browsing online, and the dates covered were stated in the References section. A final corpus of 152 studies was obtained. Figure 1 shows the selection process of the included studies. The selected literature was subsequently analysed and synthesised into schematic sections covering AI applications for pathogen detection, performance evaluation, industrial implementation, and regulatory framework. The PRISMA checklist can be found in Supplementary Materials.

## 3. Machine Learning Foundations and Applications in Food Safety

AI applications to food safety management have been increasingly explored in the scientific literature. The number of publications has risen sharply since 2024, with China and the United States being by far the leading contributors to research [18]. At a global level, AI applications to food safety systems are largely shaped by the nation’s organisation, with a marked digital divide between high-income countries (HICs) and low- and middle-income countries (LMICs). A systematic analysis of food safety applications from 2018 to 2025 [25] has highlighted evident structural differences: in HICs, AI aims to facilitate regulatory compliance, reinforce safety systems, and enhance predictive risk management, whereas in LMICs they are mainly limited to basic screening and operational risk control. In fact, 78% of LMICs have insufficient cloud infrastructures [26], which is a critical gap for AI development. This disparity is extremely important, considering that LMICs bear the most significant burden of foodborne diseases, which require urgent modernisation [27]. International bodies, like the Food and Agriculture Organisation (FAO), are promoting capacity building, aiming to ensure equitable data access and facilitate dialogue with private stakeholders [28]. Such concerted actions, combining the power and the experience of private entrepreneurs with the regulatory guidance of international institutions, will be essential for developing safer food systems in LMICs [29].

### 3.1. AI-Driven Microscopy and Image Analysis for Bacterial Detection

Bacterial cell morphology encompasses multiple dimensions of information that collectively define species identity and physiological state [30]. AI-enhanced image analysis can provide objective, consistent evaluation of these morphological features, substantially reducing classification time and eliminating operator-dependent inconsistencies. By combining AI with microscopic imaging, researchers can analyse complex bio-optical signals that traditional methods often miss, especially when dealing with pathogens under environmental stress. A recent comprehensive review by Papa et al. [31] examined the integration of AI with optical microscopy techniques and reported numerous applications for foodborne pathogen detection and identification.

AI-assisted microscopy can also detect morphological changes induced by physical, chemical or thermal treatments. Wu et al. [32] successfully distinguished between three different bacterial conditions: live *E*. *coli*, heat-killed *E*. *coli* (treated at 100 °C for 10 min), and autoclave-sterilised *E*. *coli* (treated at 121 °C for 15 min) through a hyperspectral imaging spectrometer attached to an upright microscope. Venugopal et al. [33] applied computational morphology analysis to reveal previously undetected phenotypic variation in response to environmental stressors. Through DL-based object detection combined with image classification, they demonstrated that *Lactiplantibacillus plantarum* exhibited a 41% increase in cell length at acidic pH (3.5) compared to neutral conditions (6.5), changes not previously detected through manual microscopy. This result highlights the ability of AI-assisted morphological analysis to detect subtle but biologically relevant cellular adaptations. Ali et al. [34] identified *E*. *coli* antibiotic susceptibility using microscopic imaging and AI by detecting morphological changes in bacteria caused by antibiotic exposure. In fact, when sensitive bacteria are treated with effective antibiotics, they exhibit altered physical characteristics—such as cell elongation or filamentation—and stop their replication cycle, whereas resistant strains maintain their normal shape and continue to grow unaffected.

Cell shape is one of the most readily observable phenotypic characteristics, including cocci (spherical), rods (cylindrical), spirilla (spiral), and pleomorphic forms. Colony morphology on agar media, expressed in terms of size, shape, colour, opacity, and edge characteristics, represents one of the oldest diagnostic features in classical microbiology. Some authors combined standardised smartphone photography with the Teachable Machine^®^ image analysis platform to classify Gram-negative bacterial colonies based on macroscopic morphological characteristics, including colony size, shape, colour, and surface appearance [35].

Other microscopy modalities include bright-field, phase-contrast, fluorescence, and atomic force microscopy (AFM). Bright-field and phase-contrast microscopy can also reveal bacterial motility patterns, including flagellar movement, gliding, and twitching. Table 1 summarises several microscopy techniques coupled with AI assistance.

The integration between AI and microscopy techniques relies on how different optical setups expose unique features of bacterial pathogens for the ML algorithms to process. Across the research papers, the most important aspect of this synergy is that the AI models dictate the choice of microscopy and vice versa to capture specific data dimensions—ranging from spatial to temporal to spectral domains [37,38,40].

In the field of bacterial image analysis, Convolutional Neural Networks (CNNs) have established themselves as the dominant DL architecture for image-based pathogen detection tasks, particularly in applications involving microscopic imaging, spectroscopic data, and hyperspectral analysis. Park et al. [45] presented a DL-based strategy for the rapid and accurate detection of live foodborne bacteria, specifically addressing the challenge of distinguishing bacterial microcolonies from visually similar food debris (e.g., spinach, Cotija cheese, and chicken breast). By training the model solely on bacterial images, a 24.2% false-positive rate was achieved. However, by incorporating food debris into the training data, the improved model achieved 0% false positives, eliminating misclassifications caused by food particles. Meng et al. [46] compared the efficiency and accuracy of their CNN framework, to screen single cells, against two conventional ML approaches: a k-nearest neighbours (kNN) classifier and a support vector machine (SVM). Their CNN framework successfully identified multiple cell types with over 99% accuracy using solely label-free, bright-field images, ultimately avoiding the invasive and disruptive effects associated with traditional fluorescent dyes, which can alter a cell’s lifecycle and characteristics. Therefore, CNN is described as a powerful DL architecture designed to automatically learn and recognise patterns directly from training data, removing the need for manually designing and extracting image features. Thus, large-scale, multiclass datasets can be processed effectively.

### 3.2. AI-Assisted Spectroscopic Detection of Foodborne Pathogens

Existing reviews have addressed several advanced aspects of pathogen detection technologies and their applications to food safety and clinical diagnostics [47,48,49,50,51]. Based on the literature reviewed regarding AI-assisted biosensor spectroscopy, a SWOT (strengths, weaknesses, opportunities, threats) analysis is presented in Table 2.

Wang et al. [52] reviewed the implementation of high-throughput optical sensors for food detection based on ML, also considering the exploitation of novel nanomaterials such as quantum dots, metal nanoparticles, and upconversion nanoparticles. These authors analysed different technical issues regarding signal interference, overlapping spectra, and environmental fluctuations in complex food systems and reviewed the impact of algorithms like support vector machines, random forests, and CNNs on the precise decoupling and feature discrimination of pesticide residues, heavy metals, and microorganisms.

One of the earliest reviews in this recent selection, published as a comprehensive summary by Zhou et al. [53], focused on ML methods employed in biosensing technology to improve the intelligence of food safety detection. These authors analysed technical difficulties regarding the application of classic algorithms and DL models to handle complex-structured data from electrochemical and optical biosensors. This review examines the implications on food adulteration detection, food quality prediction, and early foodborne disease warnings.

In a systematic review by Wu et al. [54], the implementation of optical biosensors for multiplexed pathogen detection has been investigated, considering also the exploitation of microfluidic devices, nucleic acid amplification technology (NAAT), and nanomaterials. The authors analysed different technical issues regarding signal crosstalk, limited multicolour label ranges, and background interference and reviewed the impact of colorimetric, fluorescence, SERS (Surface-Enhanced Raman Spectroscopy), and SPR (Surface Plasmon Resonance) platforms on rapid, on-site multi-pathogenic identification for food safety.

Fdez-Sanromán et al. [55] published a comprehensive review focusing on biosensor technologies for water quality assessment. Different technical issues emerged regarding the integration of microfluidics and the fabrication of sensors using advanced techniques like SELEX, molecularly imprinted polymers (MIPs), and self-assembled monolayers (SAMs). Moreover, this review underlines the effects of rapid detection of emerging contaminants and pathogens on public health and environmental integrity.

Spectroscopic techniques capture information on the interaction between electromagnetic radiation and matter, giving insight into sample composition and structure [56]. Raman spectroscopy provides rapid, label-free bacterial identification through analysis of characteristic molecular vibration patterns specific to each pathogenic species. Rahman et al. [57] reported that Raman spectroscopy achieves high accuracy in foodborne and clinical pathogen detection when combined with ML and DL. This technique is useful in rapid clinical diagnostics and antibiotic resistance detection by identifying antibiotic resistance markers and distinguishing between methicillin-resistant *S. aureus* (MRSA) and methicillin-susceptible *S*. *aureus* (MSSA) strains. Kang et al. [58] applied SERS with CNN for bloodstream infection pathogen detection and successfully achieved a detection accuracy of 98.68% for pathogenic bacteria (e.g., *E*. *coli*, *A*. *baumannii*, *Enterococcus faecium*, *E*. *faecalis*, *S*. *aureus*, and *P*. *aeruginosa*) and a 99.85% accuracy for identifying carbapenem-resistant *K. pneumoniae*. Abo Dena et al. [59] used an integrated system combining attenuated total reflection–Fourier transform infrared (ATR-FTIR) spectroscopy with AI algorithms to discriminate the following fungal species with 100% accuracy: *Penicillium chrysogenum*, *Aspergillus* species (*niger*, *fumigatus*, *solani*, and *flavus*), and two different strains of *Fusarium oxysporum*. To reduce the data dimensionality, the team first applied Principal Component Analysis (PCA) and then extracted the most distinguishing spectral features. These optimised features were then processed by an automated microcontroller board setup running different ML classifiers, such as SVM and kNN. The combination of ATR-FTIR spectroscopy with AI provided a simple, automated, and highly effective analytical tool for food safety, which is particularly suitable for high-throughput screening applications in seed and grain processing.

### 3.3. AI-Enabled Biosensing and Sensor-Based Detection Systems

Biosensors have become a core technology in food safety due to their ability to operate within complex chemical and biological matrices [60]. A recent systematic review carried out by Abd Rahman et al. [61] evaluated the performance, advancements, and limitations of electrochemical biosensors used for detecting foodborne pathogens, analysing data from 77 studies published between 1998 and 2025. Electrochemical biosensors are presented as portable, cost-effective, and highly sensitive alternatives to detect foodborne pathogens, such as *E. coli* O157:H7, *Salmonella* spp., and *Listeria monocytogenes*, by capturing them with bioreceptors like antibodies, aptamers, or bacteriophages and translating that interaction into a measurable electrical signal. In parallel, among the various types of biosensors used, optical sensors have emerged as rapid and interfaceable tools [62]. They work by using biological recognition elements (like antibodies) combined with functional nanomaterials (e.g., gold/silver nanoparticles, etc.) to translate the presence of pathogens into measurable optical signals, such as colour changes or fluorescence. Because colorimetric sensor results consist of visual colour changes, they are easily interpretable by a mobile phone camera [63], making them highly suitable for rapid preliminary screening on production lines or in the field (e.g., a smartphone-integrated photothermal lateral flow biosensor developed to detect staphylococcal enterotoxin A [SEA]) [64].

As optical sensors can generate complex spectral and imaging data, especially when facing interference from complex food matrices, their data integration with ML and chemometric algorithms could help process and handle complex signals by improving pattern recognition and enabling intelligent, real-time decision-making in food safety. Three primary ML methodologies are used alongside sensors [13]: unsupervised learning, supervised learning, and DL.

Unsupervised learning techniques like PCA and Hierarchical Clustering Analysis (HCA) are used to group unlabelled datasets and reduce data dimensionality, filtering out redundant information while maintaining the array’s ability to discriminate between analytes. Unsupervised learning methodologies, including K-means clustering and hierarchical analysis, facilitate pattern recognition in unlabelled datasets from complex food matrices. These approaches are particularly valuable during exploratory phases of pathogen characterisation when comprehensive labelled datasets may not exist. Self-supervised learning, leveraging contrastive learning frameworks, has shown promise in learning robust feature representations from unlabelled or partially labelled data.

Supervised learning algorithms such as Linear Discriminant Analysis (LDA), SVM, and Artificial Neural Networks (ANNs) are trained on known data to build highly accurate classifiers. These models can quickly sort new, unknown food samples into specific categories, for instance, distinguishing between different classes of parameters and anticipating the result based on the input. Traditional supervised learning remains the dominant paradigm in pathogen detection, where labelled datasets containing both positive and negative samples are used to train classification models. SVM and random forest (RF) classifiers demonstrate strong performance as baseline methods, particularly when combined with handcrafted features from spectroscopic data. However, these traditional approaches often require extensive feature engineering and domain expertise, limiting their adaptability to novel detection scenarios.

Finally, for vast unstructured datasets, such as visual colour changes in optical sensors, DL frameworks like CNNs are employed. CNNs can extract layered features directly from images, enabling precise automated predictions regarding food quality.

## 4. AI in Food Production Systems and Supply Chains

### 4.1. Meat and Poultry Processing

Food safety and compliance are key priorities in the meat and poultry industry, where AI tools can support continuous improvement of monitoring and control strategies, enabling rapid and cost-effective approaches for hazard detection. In the context of pathogen surveillance, ML is primarily applied to the analysis of heterogeneous datasets and the extraction of predictive insights from large-scale operational data [19]. Meat and poultry products remain among the highest-risk food categories for microbial contamination, particularly with *L. monocytogenes* and *E. coli* O157:H7, and especially in raw meat products [65]. Olufemi et al. [66] proposed real-time detection strategies integrating biosensors, IoT, ML, and AI within meat processing environments across the USA. Their framework relies on the deployment of sensing devices at critical contamination points, including contact surfaces, air monitoring systems, and water networks, enabling continuous acquisition of environmental parameters such as temperature, humidity, and pH, together with historical microbiological data and operational variables (e.g., sanitation schedules and equipment usage). The proposed validation methodologies involved a direct comparison between the AI predictive system’s alerts and actual laboratory results of samples taken from the facility to ensure the system’s accuracy and reliability. CNN approaches may be used to assess meat freshness [67]. Alvarez-Garcia et al. [68] explored how AI and advanced sensor technologies could help assess meat quality characteristics (e.g., colour, water-holding capacity (WHC), pH, moisture, texture, tenderness, and intramuscular fat) through non-destructive technologies such as computer vision, hyperspectral imaging (HSI), visible and near-infrared spectroscopy (VIS/NIR), magnetic resonance imaging (MRI), and ultrasonography. AI systems can also be integrated with Radio Frequency Identification (RFID) technologies and blockchain-based infrastructures to enhance traceability and ensure transparent monitoring of product provenance and storage conditions throughout the supply chain. At the farm level, RFID-enabled ear tags facilitate the continuous identification of livestock across production stages, supporting national registries from breeding to slaughter and distribution [69]. In addition, the application of ANNs helps process the data generated during food inspection, which directly improves and automates traceability efforts. Furthermore, in supply chain management, the application of AI has led to significant advances in terms of traceability and quality control.

### 4.2. Dairy Products

A growing body of literature highlights the increasing role of AI in the dairy sector, where ML, DL, IoT technologies, and blockchain systems are widely applied to improve production efficiency, logistics, and quality assurance [70]. Within this context, forecasting algorithms such as SARIMA, Tabu Search, and Ant Colony Optimisation are employed for demand prediction, inventory optimisation, and supply chain management. The increasing digitisation of dairy production processes and the availability of high-resolution operational data have further accelerated the adoption of data-driven monitoring systems. ML-based sensor arrays have demonstrated strong performance in the identification of pathogenic and spoilage microorganisms in milk. Wang et al. [71] developed a non-specific optical sensor array combining three types of two-dimensional nanomaterials (2D NPs) with fluorescently labelled single-stranded DNA (ssDNA) probes to generate distinctive fluorescence signatures for multiple bacterial species. These researchers then coupled the array with six ML models to interpret these intricate patterns. The signals were interpreted using a combination of ML models, including random forest classifiers, multilayer perceptrons (MLPs) and Kolmogorov–Arnold networks (KANs), enabling accurate discrimination among eight distinct bacterial species, including *E*. *coli*, *S*. *aureus*, and *Salmonella*. Beyond microbial identification, ML approaches have been applied to assess milk quality, purity, and authenticity, supporting both safety and fraud detection strategies [72]. At the system level, AI contributes to improved decision-making, resource allocation, and prediction of management milk production and pasture dynamics, while also enabling early detection of contamination risks and process anomalies. Dragone et al. [73] investigated a remote diagnostic system based on an ML-driven Hazard Analysis and Critical Control Point (HACCP)-like framework for dairy chain monitoring. The system could detect anomalies in milk production processes and differentiate animals based on physiological status and breed through milk composition analysis. Similarly to other supply chain applications, AI enables the identification of subtle deviations in chemical profiles, packaging integrity, and production data, thereby supporting predictive risk mapping and enhancing early warning capabilities for dairy fraud and quality degradation [74].

### 4.3. Seafood Processing and Quality Assessment

AI is transforming fish and seafood quality assessment by enabling the automated analysis of biological and quality-related attributes that were previously evaluated through labour-intensive methods. Applications include species identification, behavioural monitoring, morphometric analysis, disease detection, and evaluation of fresh quality parameters [75]. ML combined with electronic nose systems has been successfully used to assess freshness and microbial composition in marine products [76]. In addition, ML models have been applied to predict biochemical degradation indicators such as total volatile base nitrogen, trimethylamine nitrogen, thiobarbituric acid, and free fatty acids during 10-month frozen storage of filleted whiting (*Merlangius merlangus*) and Atlantic bonito (*Sarda sarda*) under different temperature conditions (−12, −18, and −24 °C), enabling the modelling of deterioration kinetics [77]. These approaches demonstrate the potential of data-driven methods to support quality monitoring, shelf life prediction, and decision-making in seafood supply chains (Table 3).

### 4.4. Fresh Produce Safety and Quality Monitoring

Agricultural and fresh produce systems increasingly rely on intelligent monitoring frameworks to ensure safety, optimise yield, and reduce post-harvest losses. ML approaches, including RF, SVM, and DL architectures, have been widely used to identify complex relationships between weather conditions, vegetation indices, and productivity patterns, enabling more accurate yield estimation [85]. The integration of IoT-based ground sensors further extends these capabilities by capturing fine-scale environmental variations, such as soil moisture dynamics and early pest infestation signals, thereby improving the granularity of decision-support information for crop management. From a food safety perspective, fresh produce contamination by coliforms and other pathogenic bacteria may occur through irrigation water, wildlife exposure, or post-harvest handling, representing a persistent risk across the supply chain. DL approaches combined with UV-C fluorescence imaging have been applied to detect *E*. *coli* contamination on citrus and spinach leaves, achieving validation accuracies above 92% in spinach [86]. Furthermore, intelligent packaging and freshness monitoring systems have been developed using 3D printing technologies integrated with lightweight Deep Convolutional Neural Networks (DCNNs). For example, dual-colour CO_2_-sensitive labels incorporating bromothymol blue and methyl red have been used to track gaseous changes during storage, providing a visual and data-driven indicator of produce quality over time [87].

### 4.5. Cross-Sector Challenges

As discussed above, the integration of AI and ML into food safety systems has emerged as a transformative approach to better manage production and non-compliance. However, despite the considerable promise of AI-driven detection and quality monitoring systems, significant obstacles persist in data standardisation, matrix interference compensation, and analytical validation that threaten their real-world deployment and regulatory acceptance across all food supply chains. Table 4 gives an overview of some cross-sector bottlenecks in food quality monitoring and AI implementation.

As observed in Table 4, matrix interferences represent a highly specific and significant challenge that varies depending on the physical, chemical, and biological properties of the food being analysed, ranging from physical variations (e.g., particle size, viscoelastic and optical properties, density, etc.) to chemical composition (e.g., various levels of fat, protein, moisture, etc.). Moreover, data standardisation and availability represent one of the most significant bottlenecks—often referred to as the “big data” challenge—in deploying AI across food supply chains. Unlike general object recognition, accurately labelled datasets for specific food quality and safety issues are rare, expensive to create, and often kept proprietary [95].

## 5. Performance Evaluation and Operational Metrics

The rapid development of AI-enabled pathogen detection technologies has expanded the criteria traditionally used to evaluate diagnostic performance. While conventional assessments have primarily focused on analytical parameters such as sensitivity, specificity, and detection limits, the increasing demand for rapid, automated, and high-throughput monitoring has highlighted the importance of additional operational considerations. In food safety applications, where pathogen detection often involves complex matrices, heterogeneous microbial populations, and time-sensitive decision-making, evaluating a technology solely on analytical performance may provide an incomplete picture of its practical utility [31]. Recent advances in AI-assisted imaging, biosensing, spectroscopic, and molecular detection platforms have demonstrated that improvements in pathogen detection can arise not only from enhanced sensing capabilities but also from more effective extraction and interpretation of information from complex datasets. By automating feature recognition, reducing the influence of background noise, and accelerating data processing, AI has the potential to improve both diagnostic effectiveness and operational efficiency [96]. At the same time, assessing the real-world value of these technologies requires consideration of factors that extend beyond laboratory performance. Reliable operation in complex food matrices, robustness to biological variability, and compatibility with existing monitoring systems for food safety are essential prerequisites for successful implementation, yet these aspects are often insufficiently addressed in proof-of-concept studies [97].

External validation is the gold standard for assessing whether a model generalises beyond its training cohort. Yet in food safety AI applications, the depth and rigour of validation vary dramatically. A systematic analysis of 165 peer-reviewed studies in food chromatography and food analysis reveals that high-fold cross-validation and Leave-One-Out Cross-Validation (LOOCV) consistently overestimate model accuracy compared to independent external test sets [98]. For example, models often report 95% accuracy on internal cross-validation but drop to 78–82% on genuinely external data collected under different analytical conditions or from different production facilities. The main weaknesses in food safety AI studies include single-site datasets, retrospective designs, incomplete reporting of reference methods, and limited workflow testing. Common reproducibility failures include incomplete method documentation (78% of studies), unavailable code or data (85%), non-standard performance metrics (68%), unreported hyperparameters (72%), missing dataset specifications (65%), and no reporting of random seed or initialisation state (55%) [99]. These issues create cascading barriers to external replication and independent verification. Public benchmark datasets are essential for reproducible food safety AI research, yet significant gaps exist. Qian et al. [100] documented that the absence of publicly available, well-curated datasets represents a major barrier to AI/ML model development in food safety. To address this, the Cornell Food Safety ML Repository was established with three cleaned and annotated datasets: (i) *Listeria* spp. presence/absence in U.S. soil samples with metadata for soil properties, geolocation, climate, and land use; (ii) *Salmonella* and *Campylobacter* presence/absence in chicken carcasses with meteorological and temporal metadata; and (iii) faecal contamination and *E. coli* concentration in watersheds with land use, water attributes, and meteorological data. The repository includes customisable scripts and LazyPredict tools for training different ML models and explicitly addresses privacy-preserving data-sharing scenarios [100].

Recent developments in multimodal AI suggest that performance evaluation should increasingly account for the ability of detection systems to integrate heterogeneous data sources, including imaging, spectroscopic, biosensing, and process-monitoring information. Such integrated approaches may improve the robustness and reliability of pathogen detection under real-world conditions, although evidence from large-scale industrial deployments and long-term validation studies remains limited [101]. In this context, AI and big data approaches mainly act on signals and datasets generated by other analytical platforms, and their primary value lies in result interpretation, data integration, automation, and decision support [102]. Table 5 provides a comprehensive comparison of different AI algorithms used for pathogen detection and the diagnosis of infectious diseases.

### 5.1. Detection Accuracy and Sensitivity

Accuracy and sensitivity are among the most critical performance metrics in pathogen detection, as they directly reflect the ability of a system to correctly identify positive and negative samples. Numerous studies report high levels of diagnostic accuracy, sensitivity, and specificity, but these performance metrics should be interpreted cautiously. In fact, the ability of AI models to maintain comparable performance across different datasets, laboratories, and real-world conditions remains a significant challenge. Factors such as data heterogeneity, class imbalance, limited representation of rare pathogens, and variability in sample preparation may substantially affect model generalisability and reproducibility. Consequently, external validation using independent datasets and standardised evaluation protocols is essential before AI-assisted pathogen detection systems can be reliably deployed in routine food safety applications [110].

Despite these limitations, recent studies have demonstrated that AI can substantially enhance pathogen detection performance when integrated with advanced sensing and imaging technologies. CNNs consistently achieve high sensitivity and specificity in imaging-based pathogen detection tasks, whereas ensemble learning approaches and SVMs often provide robust performance when training datasets are limited [111,112,113,114]. Importantly, no single algorithm has been shown to outperform all others across different applications, indicating that diagnostic performance remains highly dependent on data characteristics, sample size, and analytical objectives. Several AI-assisted detection platforms have reported exceptionally high classification performance. For example, the integration of SERS with Vision Transformer architectures enabled rapid bacterial identification from clinical blood samples, achieving accuracies of 99.3% for Gram classification, 97.6% for species identification, and 98.5% for methicillin-resistant *S. aureus* detection [115]. Similarly, AI-enabled biosensing systems combining nanomaterial-based sensors with DL algorithms have demonstrated microbial identification accuracies exceeding 98.5% in complex water samples [116]. In liquid food and agricultural water matrices, automated AI biosensing frameworks achieved prediction accuracies ranging from 80% to 100% despite environmental noise and sample variability, demonstrating the potential of ML approaches to preserve diagnostic performance under less-controlled conditions [117].

Advances in computational microscopy have further expanded the diagnostic capabilities of AI-assisted pathogen detection. Recent studies reported classification accuracies above 98% for multiple clinically relevant pathogens using transformer-based image analysis approaches, while ANNs combined with quantitative phase imaging achieved identification accuracies approaching 99.9% following repeated measurements [118]. Collectively, these findings demonstrate the capacity of AI to enhance both detection accuracy and sensitivity across diverse analytical platforms. Nevertheless, the variability of experimental protocols, data quality, and validation procedures continues to complicate direct comparisons among studies and emphasise the necessity of standardised benchmarking frameworks and independent validation datasets [110]. Therefore, reported accuracy figures need to be evaluated cautiously, as they derive from different pathogens, matrices, sample sizes, and experimental conditions. Moreover, many studies were carried out on controlled laboratory datasets and not in real-world conditions. Thus, without a comparative or meta-analytic framing, which is not a simple task, external validation with standardised protocols is still indispensable.

### 5.2. Time-to-Result and Rapid Detection Performance

Rapid detection is a critical requirement in food safety monitoring, as delays in pathogen identification may allow contaminated products to enter the food supply chain and increase the risk of foodborne outbreaks. The time required to generate actionable results has therefore become an increasingly important performance metric for evaluating AI-assisted pathogen detection systems. Traditional microbiological workflows require at least 48 h for pathogen identification and antimicrobial susceptibility testing, with some diagnostic pipelines extending to 72 h depending on organism complexity and analytical procedures. Such delays significantly limit their applicability in time-sensitive scenarios, particularly in severe infections where early intervention is essential and may result in prolonged empiric and potentially inappropriate antimicrobial therapies [119,120].

Recent technological advances integrating biosensing platforms, nanotechnology, spectroscopic techniques, and nucleic-acid-based assays have been developed to reduce diagnostic turnaround time while maintaining analytical reliability [121]. These approaches include rapid amplification methods, miniaturised analytical systems, and improved signal transduction strategies, which collectively contribute to faster detection workflows compared to conventional culture-based methods. In this respect, AI plays a complementary role by accelerating data interpretation and enabling near-real-time analysis of complex signals generated by these platforms. AI contributes to this process by automating data processing, feature extraction, pattern recognition, and result interpretation, thereby reducing analytical turnaround times and facilitating faster decision-making. Rather than functioning as a direct sensing technology. AI primarily acts as an interpretative and decision-support layer operating on data generated by upstream analytical platforms, where its principal value lies in integrating heterogeneous data and simplifying analytical workflows [102].

Evidence of these advantages has been reported across several AI-assisted sensing platforms. Yi et al. [122] developed an AI-enabled biosensing framework based on bacteriophage-mediated microscopic pattern recognition that achieved pathogen detection in diverse water samples in less than 5.5 h while maintaining prediction accuracies of between 80% and 100%. Notably, the model was trained exclusively on laboratory-generated datasets but successfully generalised to real-world samples containing previously unseen environmental noise, demonstrating the potential of AI to accelerate microbial monitoring under realistic operating conditions. Also in this case, accuracy data should be interpreted carefully, due to the difficulty in comparing reported figures.

CRISPR-based biosensing platforms have recently emerged as rapid and field-deployable tools for foodborne pathogen detection, overcoming several limitations of conventional diagnostic workflows. By integrating CRISPR/Cas systems with nucleic acid amplification and signal transduction strategies, these approaches enable fast and specific detection in complex food matrices. Importantly, recent developments highlight their potential for significantly reduced time to results, while ongoing challenges include standardisation, multiplexing capacity, and matrix-related inhibition. The integration of AI for sequence analysis and decision-support workflows has been proposed as a key strategy to further accelerate and optimise CRISPR-based food safety surveillance systems [123]. Recently, Ma et al. [124] have coupled CRISPR/Cas with multiplex fluorescent probe-based loop-mediated isothermal amplification (mLAMP) on a microfluidic platform for improving detection of foodborne pathogens. Validation with 24 inoculated and 50 real seafood samples showed that the results were highly consistent with qPCR and culture methods.

However, despite these advances, AI-assisted time-to-result performance remains dependent on the quality, stability, and standardisation of upstream analytical signals, as well as on robust sample preparation and data acquisition protocols. Variability in experimental conditions and limited large-scale validation studies still represent key challenges for the routine deployment of these systems in food safety applications [103].

### 5.3. Cost-Effectiveness and Scalability Analysis

Routine food safety surveillance requires analytical technologies capable of processing large sample volumes under economically sustainable conditions. Therefore, the value of AI-assisted pathogen detection depends not only on diagnostic performance but also on scalability and operational expenses.

AI-based systems can improve analytical efficiency by automating labour-intensive tasks such as image interpretation, colony counting, spectral signature processing, and data classification, thus reducing manual workloads and supporting higher laboratory analytical capacity [7]. These advantages are particularly relevant in high-throughput laboratories, where continuous testing and time constraints require efficient workflow management. ML offers a strong baseline return on investment by optimising process parameters and enabling risk-based monitoring. Studies indicate that ML-driven predictive maintenance can reduce maintenance costs by 18–25%, while AI-driven analytics can optimise supply chain efficiency, logistics, and inventory management by up to 95%. AI-driven predictive maintenance can also reduce downtime by up to 30% and improve forecast accuracy by 18% while cutting operational costs by 12%. In one large-scale implementation, McKinsey documented an 18–25% reduction in maintenance costs [125].

The real economic leverage, however, lies in DL and computer vision for automated visual inspection. Many companies achieve ROI in under a year through reduced errors and reallocated labour. The global AI in food safety and quality control market was estimated at $2.7 billion in 2024 and is projected to reach $13.7 billion by 2029, growing at a CAGR of 30.9% [126]. Early adopters report up to 14% total cost savings, and companies like General Mills have saved over $20 million since fiscal 2024 by using AI that evaluates more than 5000 daily shipments [127].

However, the implementation of these systems involves substantial financial investment. High-resolution imaging devices, spectroscopic instruments, biosensors, and computational infrastructure significantly increase initial costs compared with conventional microbiological methods. Additional expenses related to maintenance, software updates, and computational resources further contribute to the total economic burden [128,129].

Beyond initial investment, cost-effectiveness should be evaluated from a full-lifecycle perspective. Economic performance is not determined solely by reductions in labour costs but also by recurring expenditures linked to data acquisition, annotation, model retraining, and infrastructure maintenance. These hidden costs can significantly affect the long-term affordability of AI-based systems and are often underestimated in laboratory-scale evaluations. In addition, scalability remains a major constraint for large-scale deployment. Although AI technologies can process complex and high-dimensional datasets and improve predictive performance [130], their transition from controlled laboratory environments to heterogenous industrial food systems is not straightforward. Differences in operational settings, a lack of standardised data pipelines, and variability in analytical workflows can reduce reproducibility and system robustness at scale [131].

Furthermore, cost-effectiveness is also affected by transparency and accountability in AI decision-making processes, which may be improved by adopting standardised collections of data (benchmark datasets) and explainable AI (XAI) methods that address the black box issue of common AI models by giving explicit explanations of how decisions are made [128,130].

Thus, most AI applications in food safety and quality management remain at the experimental or pilot stage rather than full industrial implementation. This limitation is mainly associated with data variability, computational requirements, and difficulties in system integration across diverse operational contexts [132]. Finally, effective large-scale implementation depends on both technological maturity and organisational readiness. Harmonised data management, standardised analytical protocols, and adequate computational infrastructure are essential to fully realise the benefits of AI-assisted pathogen detection. Without these conditions, the advantages of AI may remain unevenly distributed across different levels of the food industry.

## 6. Regulatory Framework for AI in the Food and Agrifood Sector

In an amazingly short period of time, legislation on AI has moved dramatically from voluntary guidelines to binding legal instruments [133]. There is a clear evolution from soft law approaches like national AI strategies and voluntary guidelines to binding regulations with compliance obligations [133]. In this regulatory space, three main jurisdictional frameworks have emerged: the comprehensive risk-based approach of the European Union (EU), the sectoral and executive order-based framework of the United States and the state-controlled governance model of China [134].

### 6.1. The European Union AI Act: Governance and Regulatory Architecture

The rapid integration of AI into food production, quality control, and microbiological surveillance has outpaced the development of sector-specific regulatory frameworks. In response to this technological evolution, the EU adopted Regulation (EU) No. 2024/1689 (AI Act), establishing the first comprehensive horizontal legal framework governing AI systems across all economic sectors. As a regulation, it is directly applicable in all member states, ensuring legal harmonisation and avoiding fragmentation across national regulatory approaches.

The legal basis of the AI Act lies in Articles 16 and 114 of the Treaty on the Functioning of the EU (TFEU), reflecting a dual objective of safeguarding fundamental rights, including privacy and data protection, while ensuring the proper functioning of the internal market. Rather than regulating AI as a single technological category, the AI Act adopts a risk-based governance model in which regulatory obligations are proportionate to the level of risk associated with specific applications. This approach reflects a shift toward anticipatory regulation designed to accommodate technological uncertainty while maintaining legal certainty [135,136].

The AI Act is embedded within a broader European digital governance framework and complements existing instruments regulating data protection, digital services, data sharing, digital markets, and product liability. Together, these instruments form an interconnected regulatory ecosystem in which AI governance is structurally linked to data governance, cybersecurity, and product safety [137].

### 6.2. United States Regulatory Approach: Sectoral and Market-Based Governance

The United States has embraced a very different regulatory philosophy, that of sectoral fragmentation and market-oriented regulation rather than a comprehensive, unified legislation. This strategy creates a governance structure of industry-specific regulations, executive orders, and voluntary standards [138] rather than an all-encompassing federal AI statute. The National Institute of Standards and Technology AI Risk Management Framework (NIST AI RMF) was intended to be a voluntary guidance document, not a mandatory regulation under Executive Order 14110 (replaced in 2025) [134]. On 3 July 2025 the policy orientation switches to executive orders to boost economic competitiveness and deregulation, and to stress speeding up innovation and global technological supremacy [139].

America’s AI Action Plan (July 2025) also situates AI development in three pillars: speeding innovation via deregulation and open-source AI support; infrastructure building via permitting and semiconductor manufacturing streamlining; and global leadership via export expansion to allies and competitor containment strategies [139]. This approach emphasises technological dominance and continued protectionist policies to ensure American technological supremacy. The regulatory environment continues to be disjointed among federal agencies, with sector-specific authorities, like the Food and Drug Administration (FDA) and Federal Trade Commission (FTC), rather than a centralised AI regulator.

The NIST AI RMF is voluntary guidance for how to govern, measure and continuously monitor AI risk. It creates a framework for responsible AI that is not legally binding [140]. This regulatory fragmentation presents a serious challenge for small- and medium-sized enterprises (SMEs) and emerging market economies that wish to operate across multiple jurisdictions, considering that the requirements for compliance vary greatly across states and sectors.

### 6.3. China’s Generative AI Governance: State Control and Content Management

China has established a comprehensive regulatory framework for generative AI, with state oversight, information sovereignty and content management [141]. The Interim Measures for the Management of Generative AI Services establish a dual registration system for algorithms and AI models, necessitating adherence to in-depth safety assessments and content quality controls [142].

China’s regulatory approach strikes a balance between technological development and risk management, including registration of algorithms, safety verification of content and data security requirements [141]. This regulatory approach, however, produces an innovation–control paradox as imperatives of information sovereignty embedded in generative AI regulation and cross-border data transfer restrictions ultimately undermine open research environments necessary to build frontier AI. This means that smaller technology companies and academic researchers are hit harder, while established players in the industry, who have the resources to deal with complicated compliance rules, are helped.

The Chinese framework tackles a range of data risks throughout the generative AI lifecycle, from the possibility of rights violations in data collection to harmful outputs and vulnerabilities in storage. Chinese regulation focuses on enforcement of compliance, transparency of algorithms, and stringent data security requirements through holistic legal structures that combine statutory requirements with adaptive regulation [143]. China’s rules for generative AI, including rules for public-facing services, content management, and data security, set standards for broad AI regulation that inform global governance strategies [142].

### 6.4. Risk-Based Regulation and Implications for Agrifood AI

#### 6.4.1. European Union

A central feature of the AI Act is its risk-based classification system, which differentiates obligations according to the potential impact of AI systems on health, safety, and fundamental rights. The regulation identifies four categories: prohibited AI practices, high-risk AI systems, transparency-obliged systems, and general-purpose AI (GPAI) models.

Prohibited practices include AI systems incompatible with EU values, such as those enabling manipulation of human behaviour, exploitation of vulnerabilities, social scoring or certain forms of biometric surveillance. Although primarily targeted at law enforcement and public administration, such provisions may also become relevant in digital food environments where AI systems influence consumer behaviour through profiling or behavioural nudging mechanisms [143,144].

High-risk AI systems constitute the core regulatory tier of the Act. These systems must comply with extensive requirements covering risk management, data governance, technical documentation, traceability, human oversight, robustness, cybersecurity, and post-market monitoring (Articles 9–15 AI Act). Rather than regulating AI algorithms alone, the Act establishes obligations throughout the entire lifecycle of the system, thereby assigning primary responsibility to developers and providers. Within the agrifood sector, high-risk classification is particularly relevant when AI functions as a safety component of products already regulated under European product safety legislation. This includes automated agricultural machinery falling under Regulation (EU) No. 167/2013 and machinery governed by Regulation (EU) No. 2023/1230. Consequently, AI integrated into autonomous tractors, robotic harvesting systems, or automated food-processing equipment may become subject to the high-risk requirements whenever its malfunction could compromise human safety or product safety.

The AI Act also establishes transparency obligations for systems interacting directly with individuals. Users must be informed whenever they engage with AI systems or AI-generated content, thereby reducing information asymmetry and supporting informed decision-making. Within food systems, these obligations may apply to consumer-orientated applications such as virtual nutritional assistants, AI-supported customer services, and personalised dietary recommendation platforms.

A separate regulatory framework is dedicated to general-purpose AI models, which are designed for broad applicability across multiple domains rather than for a single predefined function. While these models may become subject to additional obligations when they present systemic risks, their relevance within food microbiology remains largely indirect. In most agrifood applications, general-purpose models are adapted or fine-tuned to support specific analytical or decision-support tasks rather than being developed specifically for food safety purposes. Nevertheless, their increasing integration into laboratory data interpretation and decision-support workflows suggests that GPAI models may become progressively more relevant as AI-assisted analytical platforms continue to evolve [145].

#### 6.4.2. United States

The United States has taken a case-by-case approach, regulating by context depending on what each AI system is used for. E.O. 14110 requires more than 100 actions by more than 50 federal entities to implement guidelines in eight broad policy areas: (1) safety and security; (2) innovation and competition; (3) workers affected by AI adoption; (4) AI bias and civil rights considerations; (5) consumer protection; (6) privacy protections; (7) federal AI use coordination; and (8) international engagement and standardisation activities. In the US, the FDA is a major player in food safety regulation. [146,147] AI can contribute to food safety by detecting contamination in real-time, modelling predictive risks and monitoring compliance, thus decreasing risks to public health. Specifically, the United States regulatory approach in the agrifood sector stresses proportionality, which means tight controls for high-risk AI applications (e.g., autonomous pest detection systems or predictive pathogen modelling) and flexibility for lower-risk applications.

#### 6.4.3. China

China follows a fundamentally different regulatory paradigm based on sector-specific ordinances rather than a horizontal framework [141]. China regulates AI through sector-specific rules under existing laws such as the cybersecurity law, data security law, personal information protection law and internet information services management measures. Different types of AI have been regulated administratively by authorities including the Cyberspace Administration of China, the Ministry of Industry and Information Technology, the Ministry of Public Security and the State Administration for Market Regulation.

This multi-authority approach mirrors the governance structure in China but has both advantages and disadvantages for regulating agrifood AI [141]. Business operators in connection with “high-risk domain AIs” must notify users that the product or service is operated based on high-risk domain AI and take measures to ensure the AI’s trustworthiness and safety. Examples of “high-risk domain AIs” include: (1) judgement or scoring AIs with a material impact on individual rights and obligations; (2) AIs used by public authorities for decision-making processes that produce legal effects or significantly impact individuals; (3) AIs used for the analysis and use of biometric data in investigations or arrests; (4) AI used for energy supply, drinking water supply, public health, medical devices, nuclear facilities, and traffic.

### 6.5. Liability, Enforcement, and Regulatory Integration

Beyond establishing technical requirements for AI systems, the AI Act strengthens the EU product safety framework by introducing explicit responsibilities for AI providers throughout the system lifecycle. High-risk AI systems are therefore regulated similarly to safety-critical products, requiring conformity assessment procedures, technical documentation, quality management systems, post-market surveillance, and CE marking before being placed on the EU market.

Liability for damage caused by AI systems remains closely linked to EU product liability legislation, which recognises software, including AI systems, as products for civil liability purposes. This approach facilitates compensation in cases involving defective AI systems while reinforcing accountability throughout the development and deployment process. However, the withdrawal of the proposed AI Liability Directive means that several questions concerning causality, evidentiary standards, and allocation of legal responsibility remain partially unresolved, leaving aspects of civil liability subject to existing national legal frameworks. Regulatory oversight is coordinated through a multi-level governance structure involving the European AI Office together with national competent authorities. Harmonised technical standards developed by European standardisation organisations are expected to play a central role in translating legal requirements into practical compliance procedures, thereby facilitating both conformity assessments and regulatory enforcement.

The AI Act should therefore be considered as one component of a broader regulatory ecosystem rather than a stand-alone legal instrument. Its implementation intersects with European legislation governing data protection, digital governance, occupational safety, and product safety, requiring AI developers and food business operators to comply simultaneously with multiple regulatory frameworks. Despite this comprehensive architecture, some uncertainty remains regarding AI systems used for predictive analytics, logistics optimisation, or supply chain management, which may exert substantial operational influence while not necessarily falling within the current definition of high-risk AI systems. This discrepancy highlights an emerging regulatory challenge as AI applications continue to expand across increasingly complex food systems.

### 6.6. AI Within the Food Safety Regulatory Framework

#### 6.6.1. European Union

Although the AI Act establishes the horizontal legal framework governing AI across sectors, its implementation within the agrifood domain must be interpreted alongside existing European food law. AI-assisted pathogen detection, predictive microbiology, and automated food monitoring systems operate within a regulatory environment primarily defined by Regulation (EC) No. 178/2002, which establishes the general principles of European food law and provides the legal foundation for food safety throughout the supply chain.

The General Food Law adopts a science-based approach founded on risk analysis, integrating risk assessment, risk management, and risk communication into a coherent decision-making framework. In this regard, AI-generated evidence may strengthen microbiological risk assessment by improving hazard identification, supporting exposure assessment, facilitating early detection of contamination events, and enhancing epidemiological surveillance. Nevertheless, AI systems remain decision-support tools and do not replace the legal responsibilities assigned to competent authorities or food business operators, whose decisions must continue to rely on scientifically validated evidence.

At the operational level, AI technologies have the potential to reinforce preventive food safety management systems based on the HACCP methodology. By continuously analysing microbiological, environmental, and production data, AI algorithms may identify deviations from critical process parameters more rapidly than conventional monitoring approaches, allowing earlier implementation of corrective actions while improving process control and operational efficiency.

Regulatory acceptance of AI-assisted analytical methods, however, depends on more than algorithmic performance. Before being incorporated into routine laboratory practice or official food control activities, AI-supported diagnostic systems must demonstrate analytical reliability, repeatability, reproducibility, sensitivity, specificity, robustness, and fitness for purpose according to internationally recognised validation procedures. Consequently, successful implementation requires simultaneous compliance with AI governance requirements and established microbiological validation standards. Recent evidence further indicates that regulatory acceptance depends on the ability of AI systems to support workflow-embedded decision-making under real operational conditions, requiring prospective, multi-site validation and demonstrating actionability within HACCP-aligned processes [99].

Alongside the legislative framework, the scientific integration of AI into the European food safety system is also shaped by the activities of the EFSA. EFSA has progressively incorporated AI into its scientific agenda, beginning with early reflections on AI applications in risk assessment [148] and subsequent roadmap development for evidence management [149]. More recently, the establishment of a dedicated AI Task Force has reinforced governance structures for human-centric AI adoption, ensuring alignment with EU regulatory principles and scientific quality standards [150]. Collectively, these initiatives confirm that AI integration within food safety remains grounded in scientific validation, transparency requirements, and human oversight, rather than replacing expert judgement.

From a governance perspective, this evolution reflects a broader transformation towards digitally enabled food safety infrastructures. As highlighted in the recent literature [27], the central challenge is not the creation of new regulatory layers but the operational integration of existing legal and scientific frameworks within data-driven, AI-enabled decision-support environments. This requires interoperable systems capable of embedding risk-based governance principles into real-time monitoring, inspection, and analytical workflows.

Overall, AI should not be regarded as an alternative to the existing European food safety model but rather as an enabling technology that complements current surveillance, laboratory diagnostics, and risk assessment activities. Its effective implementation depends on the convergence of horizontal AI regulation, sector-specific food law, validated analytical methodologies, and robust scientific evidence capable of supporting both regulatory compliance and public health protection.

#### 6.6.2. United States

AI is being integrated into U.S. food safety mainly through voluntary adoption by food companies for quality control, predictive analytics, and supply chain traceability, based on FDA guidance documents. More and more organisations, such as the Safe Quality Food (SQF) programme and British Retail Consortium (BRC) Global Standards, require or recommend AI-supported verification procedures [151]. However, there are major issues around the requirements for data quality, the regulatory validation frameworks, complexities of system integration and ethical issues. One of the biggest challenges adaptive ML models present to the FDA is that the agency must deal with unique issues in validating traditional artificial intelligence systems that change after they are deployed.

#### 6.6.3. China

In China, the use of AI in the food safety regulatory system is officially recognised as a supervision model (“Intelligent Food Safety Supervision (IFSS)”). Through the use of AI and big data, this model changes the game of food traceability, risk evaluation and law enforcement. But this alteration presents serious administrative challenges such as algorithmic biases, data silos, and the risk of private tech companies seizing democratic regulatory power. To strike a balance between technological innovation and procedural fairness, experts call for a strong legal framework to bring artificial intelligence algorithms under strict public oversight and officially regulate the participating technical entities [152].

## 7. Conclusions

AI and ML technologies have significantly improved foodborne pathogen detection by enabling faster, more automated, and more sensitive analytical approaches compared with conventional microbiological methods. In fact, the integration of DL models with imaging, spectroscopic, biosensing, and sensor-based technologies has expanded the range of available tools for contamination detection and monitoring, with surface-enhanced Raman spectroscopy demonstrating effectiveness for pathogen detection and optical sensors being applied to the interpretation of complex data. Reported high-performance results and reduced analysis times demonstrate the promise of these approaches to improve responsiveness in food safety applications.

AI-improved biosensors can help investigate elaborate setups through enhanced sensitivity, reduction of false results, and real-time applications. The combination of AI with complementary digital technologies such as IoT, blockchain, and edge computing may further support the development of integrated monitoring strategies across the food supply chain, improving traceability and information flow. In parallel, advances in explainable AI and adaptive modelling approaches are expected to enhance system robustness and applicability in complex and variable food production environments, which might be crucial for food safety risk prevention. Future convergence of AI models, ML-enhanced datasets, and rigorous validation protocols will address investigation challenges across food supply chains.

Despite these advances, several challenges remain, including data heterogeneity, limited external generalisation, a lack of standardised protocols, regulatory uncertainty, and barriers to large-scale implementation. Addressing these issues will require coordinated interdisciplinary efforts involving researchers, industry, and regulatory authorities. Furthermore, AI application to food safety systems is indeed affected by systemic capacity, with high-income countries applying AI to regulatory compliance and low-income countries facing the limitations given by insufficient digital infrastructure and limited data availability. These constraints might be overcome by enhancing national capacity and favouring collaboration with international organisations and private stakeholders.

At a global level, AI needs to be considered as an efficient decision support that does not substitute existing controls. Overall, the effective translation of these technologies into practice will depend on their ability to operate reliably within real-world food safety contexts while complementing established control and surveillance systems.

## Figures and Tables

**Table 1:** AI-assisted microscopy techniques for microbial identification.

Microscopy/ Imaging Technique	Hardware & Setup Details	AI/ML Approach	Target Features & Methodological Aspects	Example Microorganisms/Pathogens	Dataset Diversity & Sample Size	Validation	Computational Costs	Gaps & Limits	Ref.
Phase-Contrast Time-Lapse Microscopy	Nikon Ti2-E inverted microscope with Plan Apo Lambda 100× oil immersion objective, microfluidic “mother machine” traps	Video classification networks (e.g., Video ResNet R(2 + 1)D, ResNet 18)	Learns single-cell spatiotemporal growth signatures, division patterns, cell length, texture, and morphology	*Pseudomonas aeruginosa*, *E. coli*, *Klebsiella pneumoniae*, *Acinetobacter baumannii*, *Enterococcus faecalis*, *Proteus mirabilis*, *Staphylococcus aureus*	Diversity: 7 bacterial species. Sample Size: 18,101 traps (train); 1399 traps (test); 621,931 images	Tested on a separate, unseen physical experiment (1399 traps) containing mixed species	Training: 1–2 h (ResNet18) to 10 h (Video ResNet) on NVIDIA A100 GPU. Inference: 0.00744 s/sample	Trained only on lab strains; excludes Gram-negative cocci; blood samples require complex pre-separation to avoid clogging microfluidics	[36]
Hyperspectral Imaging (VNIR)	Specim Spectral Camera V10E (400–1000 nm), telecentric fore lenses, push-broom scanning in custom halogen dome	1D-CNN)	Extracts 125-dimensional “Colony Spectral Signatures” (CSS) via spatial–spectral watershed segmentation	Urinary Trait Infection (UTI) pathogens: *E. coli*, *E. faecalis*, *S. aureus*, *P. mirabilis*, *Proteus vulgaris*, *K. pneumoniae*, *P. aeruginosa*, *Streptococcus agalactiae*	Diversity: 8 UTI species. Sample Size: 16,642 colonies from 106 HSI plates	70/30 random Shuffle & Split cross-validation. No independent clinical cohort was used	Training: Fast (~1000 s) using GPU. Inference: Very fast (<0.01 s)	Near-perfect lab results reveal a need for validation in real clinical lab environments with actual patient specimens	[37]
Hyperspectral Imaging (VNIR)	Specim Spectral Camera V10E (400–1000 nm), telecentric fore lenses, push-broom scanning in custom halogen dome	1D-CNN, SVM, Random Forest combined with 3D-PMDC compression	Tests coding strength-driven compression of CSS features to provably prevent classification degradation	UTI pathogens: *E. coli*, *E. faecalis*, *S. aureus*, *P. mirabilis*, *P. vulgaris*, *K. pneumoniae*, *P. aeruginosa*, *S. agalactiae*	Diversity: 8 UTI species. Sample Size: 16,642 colonies from 106 HSI plates	70/30 random Shuffle & Split cross-validation	CNN memory footprint remains constant, while SVM/RF rises significantly as data scales	Compression operating points require careful tuning (e.g., bitplane 9) to avoid losing anomaly data	[38]
Bright-Field Optical Microscopy	Olympus CX31 Biological Microscope, SC30 camera capturing 2048 × 1532 images	DenseNet-121 (Deep Transfer Learning)	Learns spatial feature hierarchies from Gram-stained images; utilises dense block architecture	33 diverse species (DIBaS Dataset)	Diversity: 33 species. Sample Size: 689 images, artificially augmented to 5512 images	80% train, 10% validation, 10% test splits on augmented data	Training: 1 h 7 s (8.01 million parameters) on an NVIDIA K80 GPU	Dataset lacks real-world diversity; model occasionally misclassifies morphologically similar species (e.g., *A. baumannii* vs. *P. aeruginosa*)	[39]
Lens-Free Holographic Microscopy	CMOS image sensor, 532 nm partially coherent laser, automated translation stage inside an incubator	Pseudo-3D (P3D) DenseNet28 using 4-dimensional inputs (phase/amplitude, time, x, y)	Learns spatiotemporal growth signatures from reconstructed complex light fields	Coliforms: *E. coli*, *Klebsiella aerogenes*, *K. pneumoniae*	Diversity: 3 species. Sample Size: ~16,000 colonies (detect); ~9400 colonies (classify)	Blind testing on 965 colonies from 15 completely independent agar plates	Training: ~5 h (detect) + ~15 h (classify) on dual GTX1080Ti GPUs. Highly cost-effective (~$0.6/test)	Reaching the LOD (~1 CFU/L) takes up to 9 h and requires a 5 h pre-incubation step	[40]
High-Content Analysis (HCA)/Fluorescence	GE INCell 2200 high-content analyser, 100×/0.9 Plan Fluor objective, 384-well plates	Bootstrap Random Forest algorithm	Learns from extracted parameters like “Form Factor” (roundness) and localised staining intensity	*Bradyrhizobium japonicum*, *Bacillus megaterium*, *E. coli*, *Pseudomonas fluorescens*	Diversity: 3 mixed species. Sample Size: 43,709 evaluated objects	Trained on 60/20/20 splits of untreated single-strain controls, blindly applied to mixed cultures	Uses default settings in JMP Pro 14. Greatly reduces manual labour compared to CFU plating	False IDs range from 0.2% to 16.9%; mixed-culture differentiation remains early proof-of-concept	[41]
Fluorescent Microscopy	Ningbo Sunny Instruments fluorescent microscope, digital CMOS camera	Random Forest, Linear SVM, and Cross-Validation SVM	Learns from manually calculated HoG features, Hu moments, and geometric properties (e.g., Euclidean distance from point to fitting line)	*Mycobacterium tuberculosis*	Diversity: TB vs. non-TB. Sample Size: 769 positive, 1664 negative candidates	25/75, 50/50, and 75/25 split testing. No external clinical cohort	Training: ~1875 ms. Testing: ~24 ms (Random Forest)	Highly specific to rod shapes; relies on manual feature extraction rather than automatic learning	[42]
FTIR Spectroscopy	FTIR spectrometers targeting biological replicates	Hierarchical Multilayer Perceptron (MLP)	Creates spectral footprints from absorption/transmission of infrared radiation using resilient back-propagation	11 strains of pathogenic bacteria	Diversity: 11 bacterial strains. Sample Size: 1274 pixel spectra	Internal testing of the spectra	Fast convergence utilising resilient back-propagation learning algorithms	Broad categorisation succeeded, but only achieved ~75% accuracy at the precise pixel-spectra level	[43]
Raman Spectroscopy	Horiba LabRAM HR Evolution Raman microscope, 633 nm laser (13.17 mW), 300 L/mm grating, 100 × 0.9 NA objective (diffraction-limited spot ~1 µm)	1D ResNet (CNN) with 25 convolutional layers and residual connections (lacks pooling layers)	1D Raman spectra (381.98–1792.4 cm^−1^). Uses strided convolutions to preserve exact spectral peak locations; processes noisy spectra from rapid 1 s measurements (SNR ~4.1)	30 reference isolates from 22 species (e.g., MRSA/MSSA, *E. coli*, *K. pneumoniae*, *Candida albicans*)	Diversity: 30 isolates (>94% of common infections). <br>. Sample Size: 60,000 reference + 12,000 clinical patient spectra	Validated on 50 clinical isolates from patients using leave-one-patient-out cross-validation (LOOCV)	Training utilises Adam optimiser (90/10 splits). Fine-tuning evaluated over 10,000 sampling trials	SNR is extremely low; requires massive intra-sample variance datasets. True culture-free application needs highly automated sample prep for biofluids	[44]

**Table 2:** SWOT analysis of AI-assisted biosensor spectroscopy for food safety.

**Strengths**	**Weaknesses**
Ultra-sensitive and rapid detection	Matrix interference and reproducibility
Enhanced discriminative power	Heavy data dependency
Portability and field deployment	Lack of interpretability (“black box”)
**Opportunities**	**Threats**
Multimodal sensor fusion	Domain shift and transferability
Cross-device spectral standardisation	Regulatory and standardisation barriers
Explainable AI (XAI) and generative models	Cybersecurity risks
IoT and cloud integration	Nanotoxicity concerns (for nanoparticle applications)

**Table 3:** AI-ML-based technologies used for the seafood industry.

Application/ System	Primary Use Case	Technology Used	Target Seafood	Data Standardisation & Availability	Validation & Generalisability Gaps (Limits of AI/ML)	Ref.
Bionic e-Eye	Marine Toxin Detection: High-throughput portable field-test detection of marine toxins	Smartphone-based system, iPlate (iOS APP designed by Swift 1.0 and Object-C in Xcode 7), HSV colour model, ELISA	Mussels	Employs HSV colour model conversion and signal normalisation. Data can be shared dynamically via mobile internet	Validated primarily with standard toxin solutions; relies heavily on specific lighting and ELISA assay kits as a supplement to off-site lab detection	[78]
UDMOS	Fish Size Monitoring: Detecting and estimating the size of large indicator species moving underwater	Computer vision, DL (Tiny-YOLO), unsupervised modelling, motion detection	Large fishes (sharks, tunas, mantas)	Standardises processing using scaled input images. Uses open-source workflows on DataMiner cloud computing for tracking provenance	DL processing lag can miss fast-moving species without high-end hardware; motion detection triggers false positives in highly animated scenes	[79]
Shrimp-Nose	Spoilage & Shelf Life Assessment: Rapid assessment of perishable quality and shelf life	Electronic nose (6 MOS gas sensors), ML (random forest, soft-max regression, PCA, KNN)	Cultured Pacific white shrimp	Normalises sensor output voltages between 0 and 1 V. Validates algorithms through cross-validation and confusion matrices	Some algorithms (like Decision Trees) showed overfitting issues. Accurate real-world use requires mapping complex synergistic behaviours (e.g., pH and microbes)	[80]
ShrimpChain	Supply Chain Traceability: Transparent framework linking farm logs and export documentation	Public–private hybrid blockchain, smart contracts, IPFS, IoT devices, mobile apps	Shrimp	Archives multimedia data off-chain using the Inter Planetary File System (IPFS). Uses a “need-to-know” smart contract structure	Conceptual framework that faces socio-technical hurdles in Bangladesh (mistrust, skills gap). Relies on actors entering accurate data within strict time windows	[81]
Multicolour Biosensor	Freshness Assessment: Naked-eye freshness assessment based on spoilage indicators (Hypoxanthine)	Colorimetric/gold nanorods (GNRs) etching, Fenton reaction	Miichthys miiuy, whitebait	Tracks visible LSPR (longitudinal surface plasmon resonance) shifts and colour variations	Qualitative naked-eye assessments can lack the granular precision of machine-interpreted data	[82]
Paper-based Dual pH Sensors	Automated Freshness Detection: Visual and predictive freshness monitoring under refrigerated storage	Chemical sensors (methyl red & bromocresol purple), random forest ML	Mackerel, sardine, prawn, pomfret, red snapper, cuttlefish	Datasets pre-processed by handling missing values/outliers; numerical variables are normalised before train/test splitting	Needs iterative updates and hyperparameter tuning to maintain accuracy across diverse fish varieties and packaging methods. Relies heavily on manual biochemical markers	[83]
DenseNet121 Authentication App	Authentication & Freshness Grading: Distinguishing premium fish meat types to prevent fraud and grading freshness	DL (Improved DenseNet121), transfer learning, customised output layers	Salmon, rainbow trout	Applies inherited-label strategies with rigorous patch quality filtering. Uses transfer learning by freezing specific layers to optimise training	Trained on small datasets lacking external real-world validation images. Relies on subjective manual annotations. Untested against advanced fraud like artificial dyeing	[84]
Frozen Quality Predictors	Predictive Spoilage Kinetics: Predicting frozen shelf life and mapping quality degradation kinetics	ML (XGBoost, random forest, SVM, naïve Bayes, DT, MLP)	Whiting, Atlantic bonito	Input variables standardised using z-score normalisation. Synthetic Minority Oversampling Technique (SMOTE) applied for class imbalances	Highly dependent on destructive laboratory biochemical assays (TVB-N, TBA) as inputs. Lacks integration with real-time, non-destructive sensing technologies	[77]

**Table 4:** Key challenges of AI adoption in diverse food supply chains.

Supply Chain	Data Standardisation & Availability	Specific Application & Technology Tested	Validation Type (Internal/External)	Matrix Interference & Complexity	Validation & Generalisability Gaps	Implementation Constraints	Ref.
Meat	Mitigated data scarcity by using image rotation augmentation (oversampling) to balance classes and resized inputs (e.g., 64 × 64) to speed computation	Image-based meat freshness assessment (fresh, half-fresh, spoiled) using an ensemble of compact CNNs (ConvNet-18 and ConvNet-24)	Internal validation (evaluated on two public datasets, MEAT2C and MEAT3C, utilising an 80:20 training and testing split)	Spoiled meat has lower pixel intensities (darker pixels), but highly dynamic visual changes occur rapidly due to exposure to light, dust, and microorganisms	Model performance is highly sensitive to hyperparameter tuning (e.g., initial learning rate, batch size); improper tuning causes the model to diverge and fail	Pre-trained deep models (ResNet, VGG) suffer from massive parameter counts (up to 143 M) and high computational training time, necessitating compact architectures	[88]
Dairy	Used PCA to reduce variables to 5 principal components explaining 88% of variance before modelling	Predicted overall quality deterioration (desirability) during storage of spreadable processed Gouda cheese at 8, 20, and 30 °C using Artificial Neural Network (ANN) and Multiple Linear Regression (MLR)	Internal validation (laboratory-scale study utilising datasets divided into training, validation, and testing subsets split 2:1:1)	Processed cheese undergoes complex, non-linear major changes in colour, aroma, flavour, and texture during storage	The number of nodes in the hidden layer must be tightly correlated with input data amounts to prevent accuracy drops; ANNs are a “black box” lacking causal mechanism indication	MLR is strictly limited to linear relationships; ANN requires extensive weight tuning over hundreds of epochs to achieve optimal results and fault tolerance	[89]
Seafood	Sporadic pathogen presence creates extreme class imbalance and data sparsity, complicating model training	Tested an ML framework (LightGBM, XGBoost, ROSE-LASSO, SVM) utilising 12 years of spatiotemporal and environmental data to monitor and predict the detection rate and severity of pathogenic microorganisms in coastal seafood	External validation (real-world study utilising 12-year retrospective field data from the CDC and a prospective 2025 external test cohort)	Pathogen dynamics are complicated by complex non-linear relationships and spatiotemporal patterns like seasonality and geographical distribution	Restricted geographic scopes limit broader applicability, highlighting a need for cross-ecoregional data collection and global standardisation	Static binary thresholds fail under climate-driven shifts, while ML models face high computational costs or overfitting risks	[90]
Grain and Oilseed	Small dataset sizes for model training can limit the overall effectiveness of ML models across diverse food matrices	Tested a Ti3C2 MXene-based dual-mode (colorimetric & fluorescent) biosensor coupled with a smartphone-based Feedforward Neural Network (FNN) for rapid quantitative detection of zearalenone (ZEN) in corn, soybean, and peanut samples	Internal & external validation (laboratory-scale spiked recovery tests + external validation on naturally contaminated samples provided by local farmers)	Complex food matrices can cause environmental interference and false positives, necessitating dual-modal cross-validation	Models need validation across diverse consumer-grade smartphone hardware and wider food commodities to ensure broad generalisability	Scaling up the material synthesis protocol for industrial application and ensuring long-term functional stability remain challenges	[91]
Wheat and Bakery	Datasets often suffer from skewed spectral data and class imbalance, requiring robust preprocessing, normalisation, and transformations	Tested a portable Digital Light Processing (DLP)-based near-infrared (NIR) spectrometer combined with ensemble ML (Voting/Stacking, LightGBM, Extra Trees, LDA) for detecting and quantifying peanut flour contamination in wheat flour	Internal validation (laboratory-scale study utilising samples artificially blended/spiked in a controlled environment)	Physical variations like particle size and packing density introduce baseline shifts and scatter effects that obscure chemical information	Multiclass separation models often fail to validate fine-grained discrimination at very low contamination percentages	Conventional detection methods suffer from high instrument costs, lack of mobility, and an inability to process data in real-time	[92]
Nuts	Hyperspectral data is characterised by extreme high dimensionality, requiring complex spectral band selection to isolate relevant features and reduce redundancy	Tested a Squeeze-Excitation (SE) Attention-Guided 3D Inception ResNet (AGIR-3DNet) DL architecture paired with HSI for the rapid, real-time classification of Aflatoxin B1 (AFB1) contamination on almond kernels	Internal validation (laboratory-scale study using kernels artificially inoculated/contaminated in a controlled environment)	Real-world samples exhibit inherent variability in morphology, surface texture, and thickness, which significantly alters spectral signatures	Models trained on single cultivars with high artificial contamination levels and limited sample sizes may not generalise to diverse agricultural conditions	Many DL architectures are computationally intensive with massive parameter counts, preventing real-time inline industrial deployment	[93]
Fresh Produce	The accuracy of ML models heavily depends on the quality of the data and the selection of relevant features	Tested a photonics-based sensor system tracking refractive indexes combined with SVM and RF algorithms to achieve real-time detection of *E. coli* and *Salmonella enterica* on fresh produce samples	Internal validation (laboratory-scale study utilising store-bought samples that were artificially spiked with pathogens)	The performance of photonics-based sensor systems can be negatively influenced by environmental factors and sample variability	Models may not fully capture the complexity of pathogen contamination in real-world scenarios, requiring ongoing refinement	Traditional approaches to detecting pathogens involve time-consuming and costly laboratory tests that delay results	[94]

**Table 5:** Expanded comparison of AI algorithms in pathogen detection and food safety.

Algorithm Family	Compatible Data & Sensing Modalities	Core Advantages & Functional Mechanism	Matrix Interference	Cold-Chain Sample Degradation	Contamination Heterogeneity	Expanded Real-World Applications	Ref.
Tree-Based Ensembles (Random Forest, XGBoost, Gradient Boosting)	Data: Tabular structured data, clinical records, genomics, and supply chain metadata. Sensors: Spectral and electrochemical platforms	Highly resistant to overfitting through combined multi-tree voting, excelling at managing high-dimensional features and non-linear patterns	Susceptible to interference when processing raw sensor data from complex food matrices without stringent preprocessing, as real-world variance can drastically lower performance compared to controlled experiments	Can forecast spoilage outbreaks based on static tabular data but lacks the internal memory architecture to dynamically track continuous cold-chain temperature degradation over time	Relies on structured datasets that often suffer from low sample diversity, failing to capture the highly heterogeneous distribution of contaminants across different real-world batches	Detecting adulterants in milk and honey; predicting cadmium concentration in rice crops; forecasting *Salmonella* and *E. coli* outbreaks	[26,103,104]
Instance-Based & Margin Classifiers (K-Nearest Neighbours, SVM)	Data: Simple numerical arrays, chemical/spectral fingerprints. Sensors: Portable rapid tests, microfluidics, NIR/FTIR sensors	SVMs use the “kernel trick” to establish optimal boundaries in complex data, avoiding false alarms. kNN is highly adaptable with no formal training phase required	Readily disrupted by non-specific binding and background noise in complex biological samples unless signals are deeply denoised to establish clear decision boundaries	Effective for offline regressions of quality at a specific time, but less efficient than time-series models for tracking continuous degradation stages	Accuracy is limited by the “curse of dimensionality” and relies heavily on the specific sampling point, potentially missing unevenly distributed pathogens	Sorting *Listeria* and *E. coli* in dairy items; classifying meat spoilage levels via regression; detecting urea adulteration in milk	[96,103]
Convolutional & Attention Architectures (CNNs, Vision Transformers)	Data: 2D/3D images, hyperspectral scans, video streams. Sensors: Bright-field/fluorescence microscopy, visual colony counts	CNNs automatically extract deep visual features without manual input. Vision Transformers capture broad, global visual context across the entire image	Generally unaffected by chemical matrix interference, as they extract hierarchical visual features directly from raw 2D/3D images rather than chemical spectra	Can assess discrete visual stages of spoilage (e.g., colour changes in smart labels due to CO_2_ accumulation) but do not natively predict future cold-chain degradation timelines	As they primarily analyse surface-level spoilage or visible defects, they may easily miss sub-surface or unevenly distributed pathogens	Grading freshness of mushrooms and vegetable soybeans; identifying physical packaging defects on production lines; virtual, chemical-free Gram-staining of bacteria	[96,103,105]
Recurrent & Time-Series Models (RNNs, LSTMs, Multigraph NNs)	Data: Sequential tracking data, temporal sensor records, epidemiological trends. Sensors: Continuous online biosensors, dynamic gas/temperature trackers	Specifically engineered with internal “memory” to track dynamic, time-dependent evolutionary changes and progressions	Sensor inputs fed into these models can be distorted by matrix effects and complex environments, requiring accurate initial sensor readings and continuous calibration	Specifically designed with internal memory to track temporal trends, making them ideal for predicting degradation in active cold chains; however, accuracy can drop during unpredictable, sudden temperature abuse due to vanishing gradients	Rely heavily on continuous environmental sensors (e.g., gas or temperature) which monitor the bulk environment, potentially overlooking localised, heterogeneous contamination spots	Forecasting meat and salmon spoilage trajectories in active cold chains; spatial–temporal modelling to predict foodborne disease outbreaks	[26,104,106]
Generative Models & Autoencoders (GANs, VAEs)	Data: Highly sparse, imbalanced datasets, or high-dimensional raw signals. Sensors: Noisy spectral data, limited microscopic imagery	GANs generate realistic synthetic data to augment underrepresented minority classes. Autoencoders excel at unsupervised noise reduction	Autoencoders excel at unsupervised dimensionality reduction and can actively strip out noise and matrix interference from complex raw spectral data	Can synthesise data for missing time points in degraded samples, though unstable training (mode collapse) limits their reliability for real-time tracking	Generated synthetic outputs often fail to capture the true, random contamination heterogeneity of real-world environments, producing outputs that lack distinct biological meaning	Generating synthetic samples of rarely contaminated food to fix imbalanced training datasets; high-speed single-cell pathogen diagnosis using denoised Raman spectra	[96,103,106]
Natural Language Processing (NLP, BERT)	Data: Textual records, social media posts, supply chain documentation, regulatory inspection reports	Automates the rapid extraction of compliance issues, public sentiment, and supply chain anomalies directly from unstructured text	Not applicable, as these models process unstructured textual records rather than chemical food matrices	Struggles to trace physical anomalies like cold-chain degradation events across borders due to reliance on often fragmented or inconsistent supply chain metadata	Not applicable to physical contamination distribution	Mining social media for early outbreak detection; analysing supplier documents to identify counterfeit organic labels and instances of fraud	[104,106,107]
Latent Variable Regression (Partial Least Squares—PLS)	Data: Highly complex, multi-dimensional spectral patterns. Sensors: Near-infrared (NIR), FTIR, and Raman spectroscopy	Discards noisy, irrelevant variables in favour of a smaller set of “latent variables” that capture meaningful chemical patterns linking input to an outcome	Specifically designed to extract meaningful chemical patterns and discard noisy variables, but remains highly sensitive to overlapping spectral peaks in non-homogeneous matrices	Primarily a static regression technique, unsuited for dynamically tracking time-dependent degradation progressions without constant rescanning	Spectral readings capture highly localised data; therefore, a heterogeneous distribution of trace adulterants can significantly skew the bulk quantification	Accurately quantifying trace levels of sugar syrup adulteration in honey; detecting foreign additives in coconut water	[103,108,109]

## Data Availability

No new data were created or analysed in this study. Data sharing is not applicable to this article.

## Supplementary Materials

The following supporting information can be downloaded at https://www.mdpi.com/article/10.3390/foods15142562/s1, Figure S1: PRIMA 2020 Checklist.
